# Koala Genome Survey: An Open Data Resource to Improve Conservation Planning

**DOI:** 10.3390/genes14030546

**Published:** 2023-02-22

**Authors:** Carolyn J. Hogg, Luke Silver, Elspeth A. McLennan, Katherine Belov

**Affiliations:** School of Life and Environmental Sciences, The University of Sydney, Sydney, NSW 2006, Australia

**Keywords:** genomics, adaptive potential, conservation management, technological advancements

## Abstract

Genome sequencing is a powerful tool that can inform the management of threatened species. Koalas (*Phascolarctos cinereus*) are a globally recognized species that captured the hearts and minds of the world during the 2019/2020 Australian megafires. In 2022, koalas were listed as ‘Endangered’ in Queensland, New South Wales, and the Australian Capital Territory. Populations have declined because of various threats such as land clearing, habitat fragmentation, and disease, all of which are exacerbated by climate change. Here, we present the Koala Genome Survey, an open data resource that was developed after the Australian megafires. A systematic review conducted in 2020 demonstrated that our understanding of genomic diversity within koala populations was scant, with only a handful of SNP studies conducted. Interrogating data showed that only 6 of 49 New South Wales areas of regional koala significance had meaningful genome-wide data, with only 7 locations in Queensland with SNP data and 4 locations in Victoria. In 2021, we launched the Koala Genome Survey to generate resequenced genomes across the Australian east coast. We have publicly released 430 koala genomes (average coverage: 32.25X, range: 11.3–66.8X) on the Amazon Web Services Open Data platform to accelerate research that can inform current and future conservation planning.

## 1. Introduction

The field of conservation genomics is rapidly developing (Box 1), with genomics touted as a technological tool that can assist in the conservation of many species. In response to the ever-growing biodiversity crisis, a range of genome initiatives have been established to sequence all life on earth [1,2]. The global objective is to create reference genomes for either a group of taxa or all taxa within a country/landscape/ecosystem. On their own, these reference genomes may appear to have little use for conservation, yet they are a foundational tool needed to interpret variation within genes and improve our understanding of species’ adaptive potential. In a more practical sense, reference genomes and population genomics can be used to inform both policy and conservation actions [3,4]. While still in its infancy, conservation genomics has been applied to infer historical and contemporary inbreeding [5], assess mutational loads [6], and understand disease associations [7], with the aim of integrating such genetic knowledge to better protect species.

The year 2020 saw a massive change in the way many view the natural world and how science can assist in our understanding of it. From catastrophic megafires in Australia that saw 12.6 million acres burned [8] and an estimated 3 billion individual specimens lost [9] to a global pandemic that has seen over 6.5 million people die and more than 630 million people infected across 195 countries [10]. Using genome sequencing, scientists showed how COVID-19 originated in wildlife species [11] and was then transported around the globe [10]. Genome sequencing can also assist with threatened species recovery efforts, such as identifying individual relatedness to inform captive breeding programs, e.g., [12,13], population differentiation that is valuable for translocation decision making, e.g., [14,15,16,17], and maintenance of genetic diversity in threatened remnant populations, e.g., [18,19]. Genomics provides the capacity to identify genetically distinct populations where the exchange of migrants occurs so infrequently that these populations become ecologically isolated [20]. Broad-scale high-throughput sequencing also permits the assessment of functional diversity and differentiation within and between populations [21]. However, genomic data can only be a valuable resource for species conservation if samples for sequencing exist across populations of interest and the resource is made publicly available for the research and management communities.

Box 1 **Conservation genomics explained.**

The human genome project [22] led to a significant increase in sequencing technologies and commentary on the value of genome technology for conservation (see reviews [3,23]). Global consortia are now dedicated to generating genomic resources for non-model species [2]. A reference genome is the representative genome of a species that all other genomes for that species (or closely related species) can be aligned with. Think of it as the puzzle box lid, a reference guide to know where each puzzle piece goes and what its function is. Resequenced genomes are the genomes of other individuals within a species (or close relative) and are aligned to the reference genome. Sequencing coverage (or depth) plays an important role in the types of questions that can be answered using resequenced genomes [24]. If downstream analyses are assessing the adaptive potential of individuals or populations, at least 30x coverage is recommended (see [24] for further details). Another common method used in conservation is reduced representation sequencing. This method sequences only small portions (hundreds of base pairs) of the genome, with variations in these DNA reads termed single nucleotide polymorphisms (SNPs). SNPs are useful in assessing genome-wide diversity [25,26], or arguably of more importance, diversity within functional regions [27], but tend to be tailored for the neutral regions of the genome. Prior to the development of SNPs, many wildlife studies used microsatellites [28], and several continue to do so. Microsatellite marker sets are often between 10 and 30 microsatellites but tend not to be representative of genome-wide diversity [29]. Target gene capture characterizes gene families of interest and provides information regarding functional diversity [30], which can provide insight into adaptive potential [31]. As the cost of genome sequencing continues to decline, there is an increasing call for using genomic data in wildlife studies [4]. This is because once the genome is sequenced, the data can be referred to and ‘mined’ bioinformatically for years. Deep (20–30x) sequencing coverage ensures that the data obtained from finite tissue samples are captured for perpetuity. In two decades, we have moved from sequencing the human genome at a cost of USD 3 billion to being able to create an equivalent reference genome for other species at a cost of ~USD 30,000 [1] and resequencing genomes for as little as ~USD 800.

**Figure 1:**
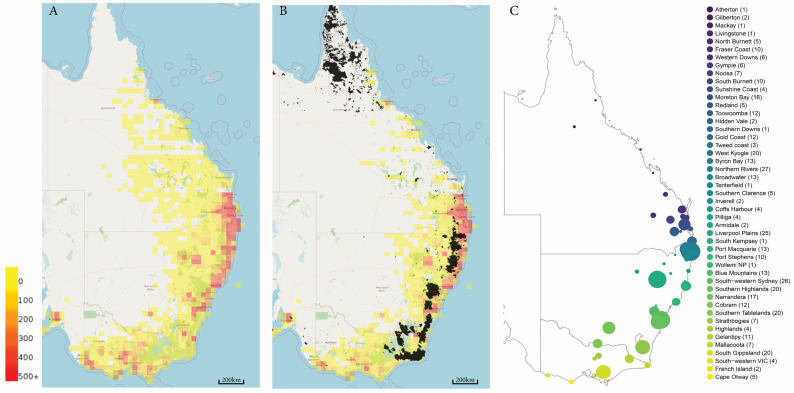
(**A**) Koala density grid from spatially valid records between 26/1/1788 and 31/12/2022 (Atlas of Living Australia). (**B**) Koala density grid from spatially valid records for the time period of samples collected for this study (1/1/2004 to 31/12/2022; Atlas of Living Australia). The black regions are the national indicative aggregated fire extent 2019–2020 (v20200324; Atlas of Living Australia). (**C**) Location of the wild samples collected as part of the Koala Genome Survey, the size of the dot is indicative of sample size, with the largest sample size being N = 20. Numbers in brackets after the location name are the sample size for that location.

The koala (*Phascolarctos cinereus*) is an iconic Australian species. This specialist folivore only eats certain types of feed trees, making their ongoing management problematic in a changing world [32], with increasing CO_2_ levels directly impacting the nutritional quality of leaves [33] and warmer climates affecting the moisture content of trees [34]. As koalas range from northern Queensland down the Australian east coast through to South Australia (Figure 1), they reside in a diverse range of habitats and feed on an array of eucalypt species, e.g., [35,36,37]. The cultural and economic value of koalas is well known, and the species has been subject to conservation planning since 1998 with the release of the first National Koala Conservation Strategy [38]. Many areas of koala habitat are in high demand for human land use through urban development and agriculture [39,40]. As a result of a changing landscape and a range of threats, including habitat loss, vehicle collisions, dog attacks, and disease, koalas were listed as ‘Endangered’ in Queensland, New South Wales, and the Australian Capital Territory in February 2022 [41]. Populations in Victoria and South Australia also suffer from these threats, and although they occur in their thousands in these states, these populations are suffering from significant historic genetic bottlenecks resulting in reduced genetic diversity [42]. 

The number of koalas in Australia is currently unknown. Although an expert elicitation on koala numbers, published in 2016, estimated there to be 329,000 (range: 144,000–605,000) koalas in Australia, with numbers declining by 53%, 26%, 14%, and 3%, in Queensland, New South Wales, Victoria, and South Australia, respectively, over the past three generations (i.e., 18 years; generation time ≈ 6 years) [43]. One of the primary causes for the ongoing decline in Queensland and NSW is wholesale land clearing and habitat loss [44]. A 2017 Australian government report found that more than half of NSW (400,000 km^2^) had experienced significant losses in ecological communities, with losses between 26% and 50% of their original extent [45]. In addition to direct impacts on koala numbers, habitat clearing increases the fragmentation of koala populations, impeding historical gene flow. This was further compounded by the significant areas of habitat that were burned during the 2019/2020 megafires [8], many of which overlay with known koala habitats (Figure 1B). Combined with other threats, it is now estimated that koalas in eastern Australia could face extinction by 2050 [46]. 

Long-term management of koalas requires an understanding of the demographic and genetic status of different populations, gene flow between populations, and current threats. Although there are many factors contributing to the management of these fragmented populations, an understanding of genetic diversity is needed to determine the capacity of a population to be able to survive long-term in this fragmented landscape or if active management interventions, such as translocation and/or captive breeding, are required. During previous periods of climate instability, koalas retracted into refugia [42,47], but it remains unclear the extent to which genetic variation is lost during short-term catastrophic events such as bushfires. After the 2019/20 megafires, we sought to understand the current genetic information that was available for the species and identify gaps in our knowledge. Although an iconic species that has been studied since the 1800s [48,49], genetic studies on koalas have only been undertaken since the 1990s, primarily using microsatellites (Table 1), with limited literature using next-generation sequencing (Box 1). Despite reduced representation sequencing being commonly used in wildlife species since 2012 [26], and the koala reference genome published in 2018 [42], there are limited examples of reduced representation sequencing in the koala literature. To better understand the potential knowledge gaps in koala genomics, we undertook a systematic review of the current literature. The aim was to understand (1) how much genetic data existed for koala populations across eastern Australia, (2) what timescale these data covered, and (3) how this may contribute to the genetic management of koalas post 2019/20 megafires. We found there to be limited population genetic studies published between 1996 and 2020 that used SNP data (3 of 24 studies; SNP range: 3060–4606 SNPs), and many studies were undertaken on the same limited number of populations. Both the NSW and Australian governments were seeking guidance in late 2020/early 2021 on how to best protect the remaining genetic diversity. To accelerate research in population genomics, functional diversity, and disease, as well as inform conservation planning for this iconic species, we established an open data resource, the Koala Genome Survey.

## 2. Systematic Review

A systemic review was undertaken in August 2020 by querying Web of Science (WoS) and Scopus using the “Topic” (WoS) and “Title” (Scopus) functions. The keywords used were ‘koala’ AND ‘genet*’ and ‘koala’ AND ‘genom*’ to obtain all genetic and genomic papers for koalas. This resulted in 551 papers that included 191 duplicates between the two search engines. Removing the duplicates left 360 individual papers. Each paper was attributed to 15 groupings (Table S1), of which 41 papers were some form of population genetic/genomic study. A full assessment of the 41 population genetic papers resulted in a further five groupings: population genetics (N = 24; Table 1); development of methods for using scat DNA (N = 4; [73,74,75,76]); DNA profiling (N = 3; [77,78,79]); differentiation of populations using mitochondrial DNA (N = 8; [52,80,81,82,83,84,85,86], and phylogenetics (N = 2; [87,88]. Of the 24 population genetics studies, two were reviews (Table 1). Several studies encompassed the whole of the koala range from Queensland to South Australia (N = 5), while others were restricted to only two states (N = 5), and 12 studies were of koalas in one specific state (Table 1). 

Population genetics papers (1996–2020) predominantly used microsatellites (N = 17; Table 1). Unfortunately, there was minimal consistency with the microsatellite markers used, with 14 studies using those developed by Houlden et al. [50] while others developed their own (N = 4), making a comparison between studies difficult [72]. Only three studies used single nucleotide polymorphisms (SNPs) from two different reduced representation sequencing methods, Diversity Arrays Technology (DArTseq; N = 2) and double digest Restriction-site Associated DNA (ddRAD; N = 1), and the reference genome paper used SNPs generated through exon capture [42]. 

## 3. Genomics and Conservation Planning

Future koala conservation management needs to address issues surrounding habitat loss and fragmentation, disease, climate change, dog attacks, and vehicle collisions via expanding and restoring habitat, vaccine development, and protecting climate refugia. If koala populations continue to decline, they will become subject to small population pressures of genetic drift, inbreeding, and loss of adaptive potential to respond to emerging threats to survival, which can ultimately lead to local extinction. Unfortunately, there is limited genetic knowledge for many koala populations across the species’ range. To make conservation planning decisions, we need to understand if populations have unique genetic variants, their level of inbreeding and relatedness, their disease status, their adaptive potential, and their effective population size. Genomics is a useful tool to address these questions and for informing conservation planning [4,89]. Looking at the number of population genetic studies in NSW, as the epicenter of the most significant megafires (Figure 1B) [8], 5 of the 10 studies published in the past six years (2014–2020; 1 koala generation) were on populations in NSW. Of these, only one study included the dates and locations of sample collection and used microsatellites to investigate koala populations in northeastern NSW [70]. Nine of 10 NSW studies have occurred within the northeastern region and within similar habitats. Expert elicitation estimated that NSW koala populations have declined over the past three generations (18 years; [43]), but we are unable to link this to changes in genetic diversity with the current data. It is similar in Queensland, where 10 of 12 studies have occurred in southeastern Queensland (Table 1). There is a wholesale lack of contemporary (i.e., within 1–2 koala generations) genetic data across the species’ range. Unfortunately, data that do exist do not have the power to provide information on genome-wide diversity (when using microsatellites) nor adaptive potential (when using ddRAD or dArTseq, see Box 1). Recently (published in 2022), two publications used target capture methods (immune genes [90] and exon capture [47]) to begin to characterize functional diversity for this species. 

A suitable conservation policy can only be implemented in the presence of sound science [91]. As is the case with many species, the lack of published and, therefore, accessible genetic data for conservation planning are limited for koalas. Less than half of published population genetic studies include the year when the samples were collected, and 45% of published studies include independent locations. Most studies include one or more sites that have previously been subject to study. For example, in NSW, this means that only six (12%) of the 49 areas of regional koala significance have contemporary (1–2 generations) genetic data that can be used to inform conservation management. Management of genetic diversity within and across populations has been linked to a species’ adaptive potential [92]. As a high-quality koala reference genome exists [42], we have the capacity to investigate genetic differences between populations, as well as functional adaptations to certain habitats and environments. Many biological functions associated with survival are the result of an interplay between a variety of genes. Understanding potential drivers associated with koala survival and gene flow can inform conservation planning in relation to habitat restoration, translocations, habitat connectivity, etc. For instance, western koala populations are experiencing more drying and significant droughts [93]. Comparative analysis of these western populations with coastal eastern populations can inform our understanding of potential genetic variants associated with ‘heat tolerance’, such as those that have been noted in Arctic charr (*Salvelinus alpinus*; [94]) and loggerhead sea turtles (*Caretta caretta*; [95]). In a similar vein, preliminary work suggests that the strength of an immune response to a *Chlamydia* infection is influenced by genetic variation within MHC Class II DMA and DMB genes and CD8-a genes [42], while MHC Class II variants are associated with infection status, serologic response, and age of presentation of *Chlamydia* disease in koala populations [96]. A recent study investigating 1209 immune genes showed that 25 SNPs across 17 genes are associated with the resolution of *Chlamydia* infection [90]. This level of information has the power to transform the way that we manage koalas in the future. For instance, as the climate changes, understanding variants involved in ‘heat tolerance’ will inform the assessment of extinction risk for populations that lack these variants. Similarly, we could reduce disease prevalence in populations by boosting immunogenetic diversity in vulnerable populations. We do not advocate management actions to promote specific variants within a population but rather promote increased genetic variation at genomic regions with low diversity, particularly regions associated with known threatening processes, such as disease [21].

## 4. Koala Genome Survey

Our solution to the current conundrum of genetic data deficiencies for koala conservation planning was to generate a publicly available resource of whole-genome resequencing data (to at least 30X coverage; [24]). We aimed to sequence up to 20 individuals per population/area to accelerate research into population differences and adaptive potential across the species’ range. Commencing in March 2021, we contacted all known koala researchers and museum collections to obtain as many samples as possible from northern Queensland to Victoria (Figure 1C). A total of 802 samples collected between 2004 and 2022 (representing the past 1–3 koala generations) were submitted for the survey. There was one sample from 1997 from an NSW location (Pilliga) that was also used due to the small sample sizes from that location. A total of 672 ear biopsies and 128 whole blood samples in EDTA were extracted using either a MagAttract HMW DNA kit (Qiagen, Hilden, Germany; cat: 67563) or a high salt method (following a modified protocol from Aljanabi and Martinez [97]). DNA concentration and quality were assessed using a Nanodrop 2000 Spectrophotometer (ThermoFisher Scientific, Waltham, MA, USA), 0.8% agarose gel electrophoresis for 30 min at 90 V, and quantified using a Qubit 2.0 Fluorometer (ThermoFisher Scientific, Waltham, MA, USA). 

Initial sequencing yielded poor results, likely due to DNA quality and library pooling. As a result, DNA repair using an FFPE DNA repair protocol (New England Biosciences, Ipswich, MA, USA) improved DNA quality. Sequencing was undertaken at the Ramaciotti Centre for Genomics (University of New South Wales, Sydney, Australia) on an Illumina NovaSeq 6000, using a TruSeq DNA PCR free library prep kit (Illumina, San Diego, CA, USA). Forty-eight samples were pooled across one lane of an S4 200 cycle flowcell. Coverage was assessed after the first sequencing run, and pooling was adjusted accordingly to meet the 30X coverage goal. A total of 430 (413 wild and 17 individuals from captive trios) samples were sequenced across 48 wild locations (Figure 1C) and two zoological institutions with an average sequencing depth of coverage 32.25X (range: 11.3–66.8X). Only 3% of the samples sequenced were collected prior to 2011. Fastq files were aligned to the koala reference genome (GCA_002099425.1_phaCin_unsw_v4.1 [42] using the Dragen Platform (v 3.8.4, Illumina San Diego, USA). After each sequencing run of 48–96 samples, data files (fastq and BAM; 58.9 TB for 430 genomes) were publicly released on Amazon Web Services Open Data program (https://awgg-lab.github.io/australasiangenomes/species/Phascolarctos_cinereus.html). A total of 430 genomes were released in September 2021 (N = 116), March 2022 (N = 144), and October 2022 (N = 170), under an open-access licensing agreement (see webpage). Metadata for each sample sequenced and released, includes sampling location, date, sex, estimated age (if known), name and contact details of sample provider, and permits that samples were collected. Other researchers who are interested in using the data and require more metadata are encouraged to contact the sample providers to facilitate research engagement and potential collaboration.

This open data resource will now be used by teams of researchers across the globe to investigate key genetic questions pertaining to koala management, including population differentiation, signatures of selection, populations at extinction risk, genetic basis of diseases such as koala retrovirus and *Chlamydia*, genetic variants associated with climate conditions and habitat types, taste receptor variation and feed tree preferences, and more.

## 5. Conclusions

Koalas are one of the most iconic globally recognized species. Even before the catastrophic megafires, many koala populations across the northern part of their range were declining due to a range of threats that will continue to be exacerbated by a warming climate. It has been predicted that Australia will continue to experience more drying and significant droughts. By creating an open data genomic resource across eastern Australian koala populations, we have generated an asset that will inform current conservation planning and be a future resource to assess whether conservation actions improve/maintain/lose genetic variation across the species’ range over time. The power of genomic data is fully realized, and with ever-declining sequencing costs, the opportunity to apply this technology to threatened species is increasing, as seen by whole-genome resources for kākāpō (*Strigops habroptilus*; [6]), hihi (*Notiomystis cincta*; [5]), killer whale (*Orcinus orca*; [98]), and Pyrenean desman (*Galemys pyrenaicus*; [99]). By generating the Koala Genome Survey, we have provided a foundational tool to protect this iconic species for future generations and provide a pathway for others to follow in generating open genomic data solutions for biodiversity conservation. 

## Figures and Tables

**Table 1:** Population genetic papers studies published between 1996 and August 2020, including location of sampling, sample collection year, and data type used in the study.

Publication Year	Reference	Location	Sample Collection	Data Type
1996	Houlden et al. [50]	NSW and SA	Unknown	6 microsatellites
1996	Houlden et al. [51]	QLD, NSW, VIC, SA	Unknown	6 microsatellites [50]
1997	Taylor et al. [52]	VIC	Unknown	6 minisatellite probes
1998	Fowler et al. [53]	QLD, NSW, VIC, and SA	Unknown	20 randomly amplified polymorphic DNA (RAPD)
2000	Sherwin et al. [54]			Review
2001	Seymour et al. [55]	NSW, VIC, and SA	Unknown	6 microsatellites [50]
2009	Cristescu et al. [56]	VIC and SA	2002–2006	15 microsatellites inc. [50]
2010	Cristescu et al. [57]	VIC and SA	2002–2006	15 microsatellites inc. [50]
2010	Lee et al. [58]	NSW	1998–2008	17 microsatellites inc. [50,56]
2010	Lee et al. [59]	QLD	Unknown	6 microsatellites [50]
2012	Lee et al. [60]	QLD	Unknown	6 microsatellites [50]
2012	Lee et al. [61]	VIC	2008–2009	12 microsatellites inc. [50,56]
2013	Dudaniec et al. [62]	QLD	2006–2009	6 microsatellites [50]
2013	Lee et al. [63]	NSW	Unknown	6 microsatellites [50] and mitochondrial DNA
2014	Ruiz-Rodriguez et al. [64]	QLD	Unknown	14 microsatellites (new ones developed)
2014	Seddon et al. [65]	QLD	Unknown	6 microsatellites [50]
2016	Kjeldsen et al. [20]	QLD, NSW, VIC, and SA	Unknown	ddRAD (3060 SNPs after filtering)
2016	Dennison et al. [66]	NSW and QLD	Unknown	14 microsatellites (new ones developed)
2016	Ruiz-Rodriguez et al. [67]	QLD and VIC	Unknown	13 microsatellites inc. [64] and mitochondrial DNA
2018	Wedrowicz et al. [68]	QLD, NSW, VIC, and SA	2013–2016	12 microsatellites inc. [50,56]
2019	Kjeldsen et al. [69]	QLD, NSW, VIC, and SA	Unknown	DArTseq (4606 SNPs after filtering)
2019	Norman et al. [70]	NSW	2012–2015	17 microsatellites inc. [50,56,66]
2020	Schultz et al. [71]	QLD	2013–2017	DArTseq (427 SNPs filtering)
2020	Seddon and Schultz [72]			Review

## Data Availability

Data is publicly available at https://awgg-lab.github.io/australasiangenomes/species/Phascolarctos_cinereus.html.

## Supplementary Materials

The following supporting information can be downloaded at: https://www.mdpi.com/article/10.3390/genes14030546/s1, Table S1: Systematic review groupings of different publications.
