# Supplementary material for: Koala Genome Survey: An Open Data Resource to Improve Conservation Planning
Source: Genes (Basel). 2023 Feb 22;14(3):546. doi: 10.3390/genes14030546 (PMC10048327; doi:10.3390/genes14030546)
Supplement: Supplementary file 1 [file genes-14-00546-s001.zip › genes-2197796-supplementary.pdf]

genes

Table S1: Systematic review groupings of different publications

Systematic review grouping	Number of publications within each group
Captive koalas	3
Chlamydia	60
Conservation	6
Disease	25
Genome	4
Habitat	6
History of koalas	3
Immunogenetics	16
Metabolism	4
Microbiome-Diet	6
Population genetics	41
Reproduction	19
Retrovirus	55
Stress	1
Other	110
* Humans	28
* Marsupials	8
* Wildlife	45
* Miscellaneous	8

* All grouped as 'Other'
